# Scale-Free Music of the Brain

**DOI:** 10.1371/journal.pone.0005915

**Published:** 2009-06-15

**Authors:** Dan Wu, Chao-Yi Li, De-Zhong Yao

**Affiliations:** 1 Key Laboratory for NeuroInformation of Ministry of Education, School of Life Science and Technology, University of Electronic Science and Technology of China, Chengdu, China; 2 Center for Life Sciences, Shanghai Institutes for Biological Sciences, Chinese Academy of Sciences, Shanghai, China; University of East Piedmont, Italy

## Abstract

**Background:**

There is growing interest in the relation between the brain and music. The appealing similarity between brainwaves and the rhythms of music has motivated many scientists to seek a connection between them. A variety of transferring rules has been utilized to convert the brainwaves into music; and most of them are mainly based on spectra feature of EEG.

**Methodology/Principal Findings:**

In this study, audibly recognizable scale-free music was deduced from individual Electroencephalogram (EEG) waveforms. The translation rules include the direct mapping from the period of an EEG waveform to the duration of a note, the logarithmic mapping of the change of average power of EEG to music intensity according to the Fechner's law, and a scale-free based mapping from the amplitude of EEG to music pitch according to the power law. To show the actual effect, we applied the deduced sonification rules to EEG segments recorded during rapid-eye movement sleep (REM) and slow-wave sleep (SWS). The resulting music is vivid and different between the two mental states; the melody during REM sleep sounds fast and lively, whereas that in SWS sleep is slow and tranquil. 60 volunteers evaluated 25 music pieces, 10 from REM, 10 from SWS and 5 from white noise (WN), 74.3% experienced a happy emotion from REM and felt boring and drowsy when listening to SWS, and the average accuracy for all the music pieces identification is 86.8%(*κ* = 0.800, P<0.001). We also applied the method to the EEG data from eyes closed, eyes open and epileptic EEG, and the results showed these mental states can be identified by listeners.

**Conclusions/Significance:**

The sonification rules may identify the mental states of the brain, which provide a real-time strategy for monitoring brain activities and are potentially useful to neurofeedback therapy.

## Introduction

Understanding the most mysterious brain activities is a long-term goal of science. Many technologies, including high-density electroencephalogram (EEG), functional magnetic resonance imaging (fMRI), and magnetoencephalography (MEG), have been developed in recent years to address this goal. With the exception of EEG, however, most of these techniques are spatially restricted and cannot be used in real-time. Furthermore, the obtained information is almost always presented as complicated visual images or waveforms. The EEG is a real-time process; if low-frequency brain waves could be heard after translation by a special sonification rule, we may be able to directly “perceive” brain activity and its variations using our auditory system. As the frequency range of human hearing is large (ranging from 20 Hz to 20,000 Hz) and the average person can hear subtle differences in frequency, the hearing strategy may provide not only real-time monitoring of brain activities but also a more sensitive way to detect the small variations in the amplitude and duration of brain waves that are ignored by conventional EEG technique.

To hear the hidden brain activities from an invasive scalp EEG has long been a dream of neuroscientists. The earliest attempt to hear brainwaves as music was made in 1934 [1]. A “Music for Solo Performer” was later presented in 1965 [2], and other similar music pieces followed. In most of these early works, however, only the amplitude of the alpha waves or other simple and direct characters from EEG signals were utilized as the driving sources of the musical sound. In the 1990s, various new music generating rules were created from digital filtering or coherent analysis of EEG [3]. According to the utilized EEG features, the techniques may be classified into two categories. The first category includes the parameter mapping method [2], [4], [5], which translates a few parameters of EEG to the characteristic parameters of music, and the event triggering approach, which utilizes specific events such as interictal epileptic discharges as triggers for the beginning of music tones or other sound events [6]. The second category is the musical application of Brain Computer Interface (BCI) [7].

The above sonification rules are mainly based on an inherent assumption that EEG signal arises from an approximately stable linear system. However, in the most recent twenty years, the assumption that the human brain is a complex system has been widely confirmed by scale-free phenomena, such as the power law or Zipf's Law. In fact, power law exists at almost all levels of neural physiology, including that of ion-channels [8], interspike intervals (ISI) [9], populations of local field potentials (LFPs) [10], scalp EEGs [11], functional networks of the brain [12], and behaviors of human society [13]. Meanwhile, many studies have revealed that all sounds (music) [14]–[17] and images [18] that humans find enjoyable are composed of scale-free structure. Therefore, in the computer music fields, composers began to write music in terms of the fractal structures with algorithms. The scale-free algorithm for generating pitches was first described by Martin Gardner [19] and has become a standard in algorithmic music [20]. These facts imply that the human brain may have adapted to the scale-free natural environment during evolution and run in a scale-free manner. Such an intrinsic universal trait may have appeared even before language [21].

If brain waves can be translated into sound by some algorithms, what could we hear from an EEG? In addition to the various linear properties of the EEG, the intrinsic scale-free phenomenon should also be embodied within its sound. In the current study, we proposed sonification rules between the characters of the EEG and the musical notes, which include the mapping method from EEG waveform amplitude to notes pitch on the basis of the scale-free phenomenon, the change of EEG power energy to notes volume according to the Fechner's law, and the period of EEG to the notes duration. To test our music generating strategy, a few EEG segments have been converted into music pieces and evaluated by listeners.

## Methods

### From EEG amplitude to pitch of musical note

In this study, we established a sonification rule between the amplitude (*Amp*) of an EEG waveform and the *Pitch* of a musical note (the logarithm of frequency), 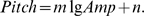
(1)The derivation of equation (1) is as follows.

In detrended fluctuation analysis (DFA) of EEG [22], suppose that *y(t)* denotes the original EEG time series with the discrete time t from 1 to T. Divide the interested range of t into B equal windows of size *k*, and let 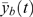 denote a straight line obtained from a least-square fit of *y(t)* in the b^th^ window, *F(k)* denotes the root mean squares of the fluctuation *y(t)* from 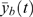 with b from 1 to B. Then, the dependence of *F*(*k*) on the window size *k* exhibits a power-law behavior [22], [23]. 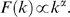
(2)Further, if the data acquisition rate is denoted by *r*, then the frequency 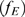 of the EEG signal corresponding to *k* is 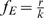 [22]. Therefore, 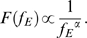
(3) Here, 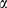 is an exponent index that usually varies between 1 and 2, and 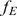 usually ranges between 0.1 Hz and 40 Hz. By performing a logarithmic operation on both sides, we have 
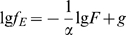
(4) where *g* is a constant. According to Fechner's law [24], the relationship between music 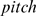 and the frequency 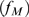 of an instrument is 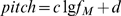
(5) where *c* = 40 and *d* = −36.6 In the MIDI standard, there are 128 pitch steps with semitone intervals among them. In this work, we choose the usual 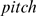 range [24 108] with the frequency 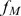 in the range [33 4186] Hz.

Human hearing ranges from a frequency of 20 Hz to 20 kHz; that is entirely different than the frequency range of EEG. There may be a specific transform mechanism between high frequency input information and the internal working frequency in the brain. For example, the human auditory system developed a function to find the “missing fundamental” [25], when humans hear only high frequency harmonics, they can feel the lost foundation frequency as well. In this way, though the human brain may not be able to hear EEG directly, it may hear the harmonic sound. Without a detailed mechanism, we assume that there is a functional relationship between 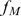 and 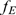. We use the simplest linear approximation 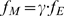 With 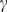 as the proportional coefficient in this work. With equations (4)–(5), we have 
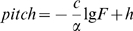
(6) where *h* is a constant. According to the definition of *F*, it is related to the change of amplitude. We may thus assume that there is an approximately proportional relationship between *Amp* and *F.*

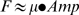
(7) Here, 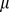 is a constant greater than 0. In the following implementation of music, the *Amp* (peak-to-peak value) is measured using the zero-cross method [26]. Based on equations (6)–(7), equation (1) is obtained with *n* as a constant and 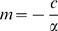
(8) From equations (1) and (8), 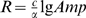 give us the dynamic range of pitch. In practice, for any known dynamic system with a power-law exponent index *α*, we can calculate this value *R* first and then reshape it to a proper region of the MIDI (musical instrument digital interface) pitch range. We assume that each individual wave matches a note. We usually take the maximal value of amplitude *Amp = *200 µV, and the *α* values of EEG range from 0.5 to 1.5 [11]. In this work, unit µV was took directly, because the only difference for different unit is the constant *n* in eq.(1). If we choose *α* = 1.10 and with c = 40, we have R = 84; thus, the pitch may vary from 24 to 108 in the range of the 128 pitch steps in MIDI. Furthermore, the DFA of EEG showed that there are two scale-free regions with two different scaling exponents *α* [22], [23]. We may therefore have two different *α* and *m* for the corresponding EEG signals.

### Mapping rules for other variables

**Figure 1:**
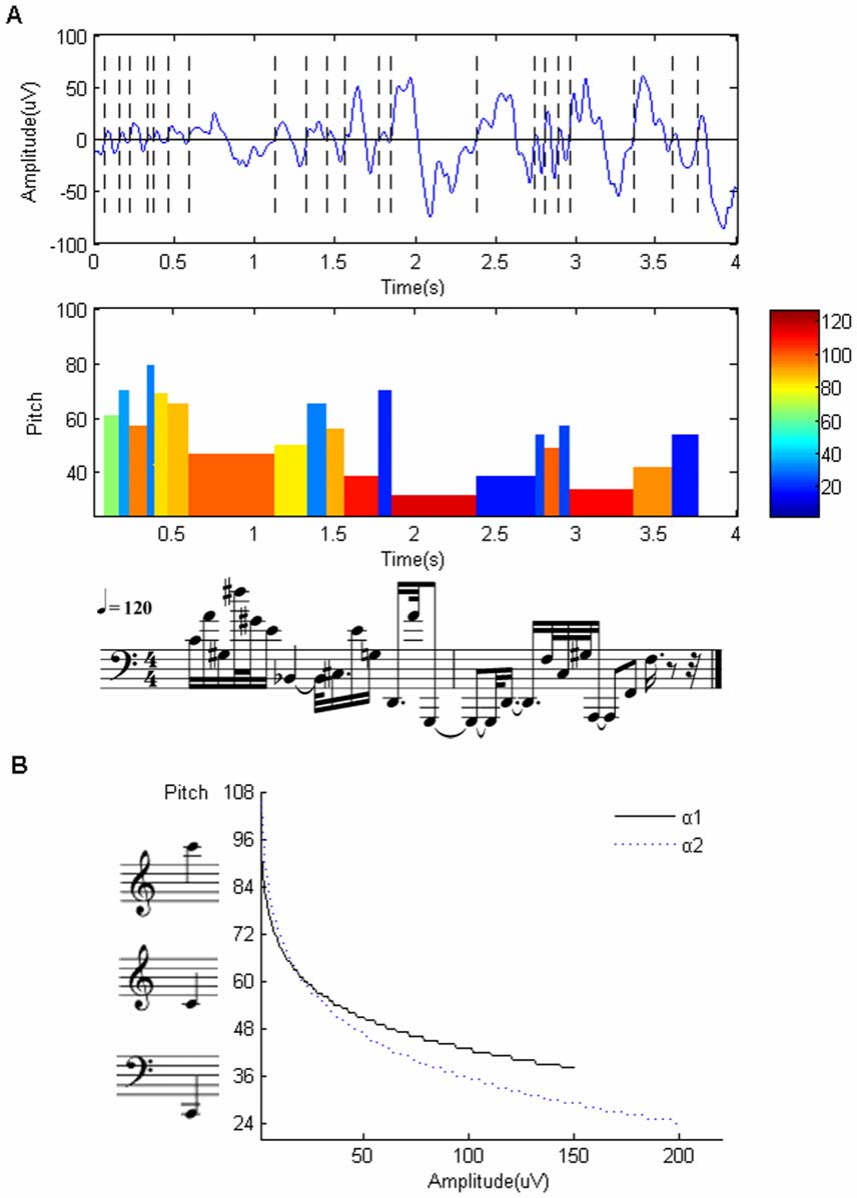
EEG and music mapping rules. A. The mapping method. B. The mapping rule of pitch. Each EEG wave matches a note. The pitch of the note is determined by equation (1), and the duration of the note is defined as the period of the wave. In part A, the top graph shows raw EEG data. The middle graph shows the pitch (height of the columns) and duration (width of the columns) of each note. The intensities of the notes are indicated by color. The bottom graph shows the musical notes derived from the EEG. Part B plots pitch value as a function of EEG amplitude. The curves are derived from equation (1) with *c* = 40, *n_1_* = 96, *n_2_* = 108, 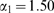, and 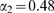; from equation (8), 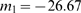 and 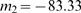. Because the pitches here are stepped by half-tones, the curve exhibits inflexions.

A musical note consists of four essential characters: pitch, timbre, duration, and intensity. Equation (1) gives the mapping rule for pitch. The timbre may be fixed by choosing a particular musical instrument, such as a piano in this work. The duration of a note may be defined as the period of an EEG waveform, and the music intensity (*MI*) of the note may be assumed to be proportional to the logarithm of the change rate of the average power (*AP*) according to Fechner's law with the formulation (9). 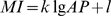
(9) Such a definition is based on the psychological fact that stimulus information may not be efficiently conveyed by a habitual signal but by a change, such as the P300 component evoked in an oddball experimental paradigm in event-related potential study [27]. Figure 1 shows the mapping rules for the three essential music characters we propose.

### Experiments

To demonstrate the performance of the proposed mapping rules, we applied this method to real EEG data. All the EEG recoding experiments and the music evaluation tests were conducted according to the principles expressed in the Declaration of Helsinki, and were approved by the Institutional Review Board of University of Electronic Science and Technology of China. All subjects provided written informed consents for the collection of samples and subsequent analysis. For sleep EEG data, recorded during the rapid-eye movement sleep (REM) and slow-wave sleep (SWS), the subject was a 25-year-old male, physically and mentally healthy, right-handed. The signals were recorded by a 32 channel NeuroScan system with a sampling rate of 250 Hz and were band-pass filtered from 0.5 Hz to 40 Hz. The data is referenced to infinity [28], and the following analysis was performed on the data at electrode Cz. The data for music generation is acquired from the second night of the subject sleeping with the braincap.

**Figure 2:**
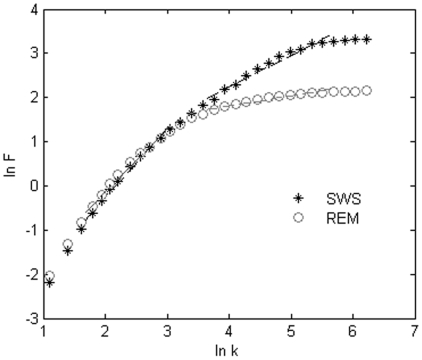
Power-law of sleep EEG with the DFA method. Here *k* is the window size, and *F(k)* is the fluctuation from the local trends in windows with *k* time points [17]. For REM, 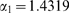 and 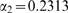; for SWS, 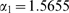 and 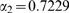.

Figure 2 shows the scale-free behavior (equation (2)) of the EEG signals. There are two scale-free regions with two different scaling exponents. The boundary between the two regions is at the alpha wave frequency range [22], [23]. For the higher frequency, the *Amp* is usually small [29]. The two 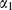 are 1.4319 (REM) and 1.5655 (SWS). We take 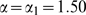 and show the result in Figure 1A for the corresponding 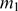 of −26.67. For the frequency region lower than the alpha wave, the *Amp* is usually large [29]. The two 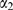 are 0.2313 (REM) and 0.7229 (SWS). We take 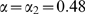 and show the result in Figure 1B for the corresponding 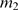 of −83.33.

### Evaluation Test

In order to ascertain if the scale-free music of different sleep states can be identified, and to see what the main feeling is when people listen to them. 60 healthy students participated in this test (41males, 19 females), ranging in age from 18 to 25 years (mean 21.05, SD 2.36). None of the volunteers reported any neurological disorders, psychiatric diseases, or were on medication. All had normal hearing. 95% of them were without special musical education, and 5% of them had special musical training less than 2 years.

We designed a test with 25 music pieces consisted of 10 from REM, 10 from SWS and 5 from white noise (WN, Audio S3) with the same mapping rule. Each music piece lasted 30 seconds and they were randomly played to the volunteers.

The test consisted of three steps: first, the volunteers were instructed to listen to two music pieces A (REM music) and B (SWS music), and focus on the differences between them. They did not know where the music was from. After the display, they were required to mark the differences between A and B of pitch, volume, tempo and timbre. Second, the volunteers were arranged to listen to the 25 music pieces in a stochastic order. After each music piece was played, they were asked to identify whether the listened piece was similar to A, B or neither. During the experiment, if someone felt he forgot the feelings of A and B, we gave him one more chance to re-experience A and B. If the music piece was from REM, and the listener thought it more like piece A (REM music), it was defined as “correct”, while the listener thought the piece more like B or neither A nor B, it was defined as “incorrect”, and same way for music B. Third, after the second step finished, they were required to write down their general feelings of the three kinds of music.

## Results

### Music of sleep EEG

**Figure 3:**
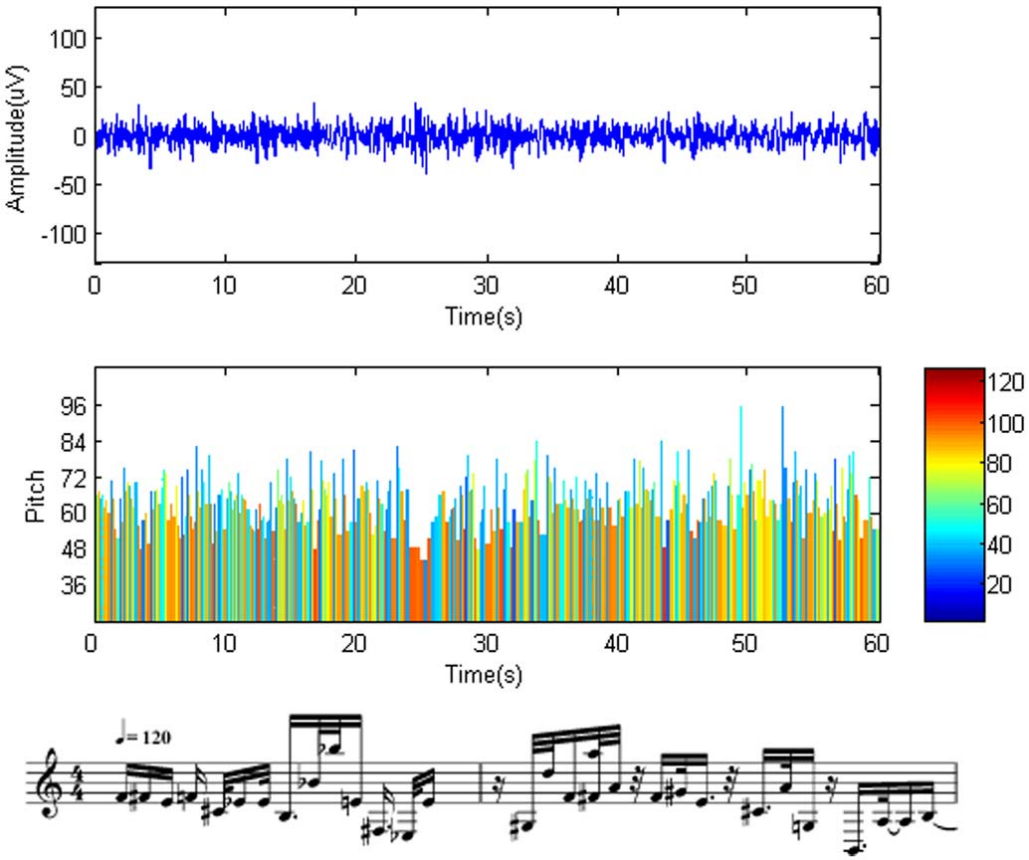
Musical notes obtained from REM sleep state. Top trace: original brainwaves. Middle trace: the corresponding notes translated from the brainwaves. The vertical columns represent the pitch (height of the columns), duration (width of the columns), and intensity (color of the columns) of the notes. The same time scale is used in the top and middle graphs. Bottom trace: Musical notation obtained from the EEG segment of the beginning. The staves are from MIDI sequences with a tempo of 120 beats per minute.

**Figure 4:**
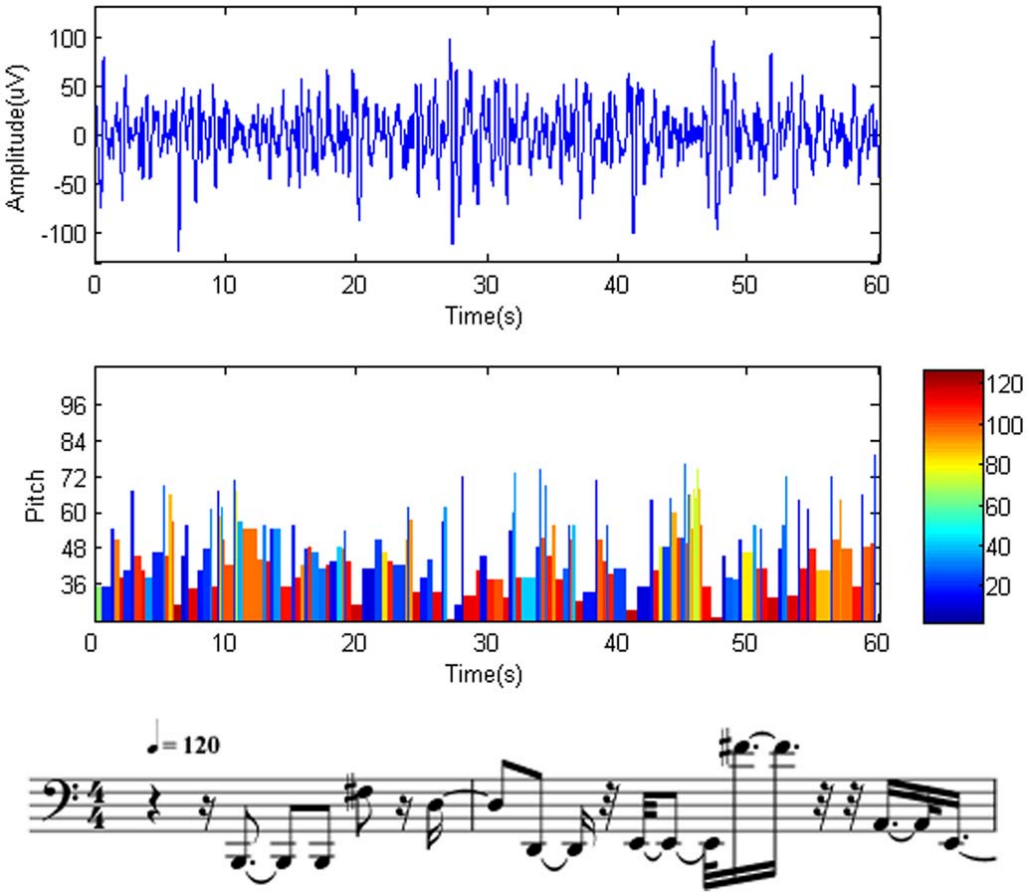
Musical notes obtained from SWS sleep state. Top trace: original brainwaves. Middle trace: the corresponding notes translated from the brainwaves. The vertical columns represent the pitch (height of the columns), duration (width of the columns), and intensity (color of the columns) of the notes. The same time scale is used in the top and middle graphs. Bottom trace: Musical notation obtained from the EEG segment of the beginning. The staves are from MIDI sequences with a tempo of 120 beats per minute.

**Figure 5:**
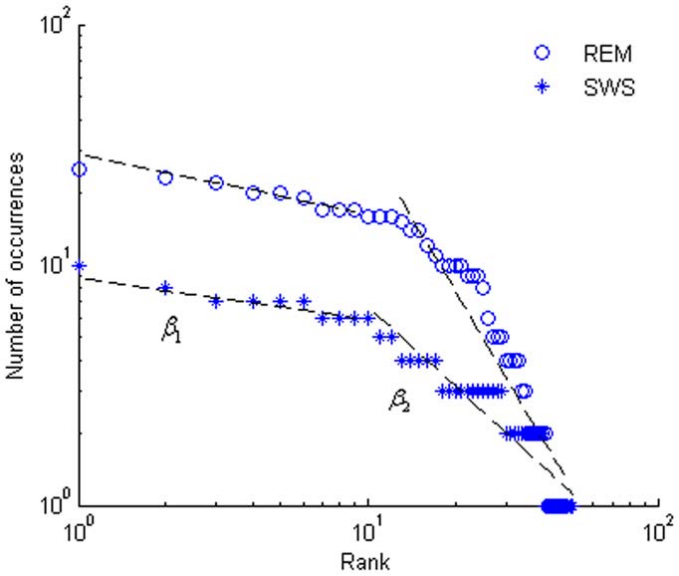
Zipf's law of music. The pitch distributions of REM and SWS musical pieces. The rank and value of the pitch occurrence number are plotted on the horizontal and vertical axes, respectively. The two different symbols represent the corresponding music pieces. The trend lines are based on the marks of each kind. For REM, 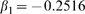 and 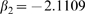; for SWS, 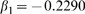 and  = −1.2478.

The resulting music pieces are shown in Figure 3, 4. The results demonstrate that REM music (Audio S1) encompasses a wide variety of note pitches. The fast rhythm and lively melody (Figure 3) suggest an active state of the brain in REM. On the contrary, the SWS brainwaves are characterized by a larger amplitude and longer duration, which results in a piece of music (Audio S2) dominated by low pitches and a slower rhythm (Figure 4). It sounds more like a lullaby, which fits nicely with the fact that the brain in SWS is in a tranquil and relaxed state [30]. Interestingly, the pitch distribution of our brainwave music is demonstrated to follow the Zipf's law [16] (Figure 5). This means that the present method retains the scale-free properties of the EEG data and that the values of exponent index are all within the reasonable range of music [16].

### Music evaluation

In the first step, the ratios of subjects who found differences between A and B in pitch, tempo, volume and timbre are 76.7%, 98.3%, 53.3% and 13.3% respectively. These results indicated that the differences in pitch and tempo are larger than that of volume. The possible reason may be that the pitch and tempo are related respectively to the EEG amplitude and period, and they are quite different between the two sleep stages. In addition, the timbre was assumed the same for all cases, thus the volunteers could not discriminate them well.

The average identification accuracy of all the music is 86.8%, and the kappa statistical parameter *κ* that estimates the overall agreement is significant (*κ* = 0.800, P<0.001). Table 1 shows the details of the identifications of REM, SWS and WN music.

**Table 1:** Matrix of the identifications results of 25 music pieces listened by 60 volunteers.

	A	B	Other	*Accuracy*
A (REM)	518	6	76	86.3%
B (SWS)	20	492	88	82.0%
Other (WN)	6	2	292	97.3%

The total tests of A, C and WN are 600, 600 and 300, respectively. The totally average accuracy is 86.8%.

About the general feelings of A, B and WN, the final written notes indicated that 78.3% of the listeners thought that A has higher pitch range, faster tempo than B, and 73.3% of them considered that A could deduce a happy emotion while B could be boring and drowsy.

## Discussion

Except the above sleep EEG, we also tested the method on EEG from eyes open or eyes closed of a 25 years old male student, and interictal EEG of a 30 years old male epileptic patient. The music pieces can be found in the supporting materials. Exposing the music to the listeners, the correct ratio for the identification of eyes closed music (Audio S4) and eyes open music (Audio S5) is 75.6%. They felt that the music for eyes closed were faster than eyes open, and the reason may be due to the dominant alpha wave during the eyes closed condition. For the epileptic data, the EEG usually contains some specific bursts of large amplitude consisted of low frequency components, therefore, some notes with long duration would appear when the epileptic specific activity occurred (Audio S6), and that make it easy to identify and differentiate it from others.

Compare to the widely adopted spectra based method [4], [6], there are some distinct differences: 1) In our method, two psychological or physiological laws are adopted, the power law for pitch and Fechner's law for volume, and these two rules are non-linear, while the rules defined in spectra based method are usually based on the linear characteristics of EEG; 2) Our method is more convenient to realize on –line because we adopted a strategy of each wave constituting a single note; 3) About the frequency information, as we adopted the “period of a wave” for the “duration of a note”, we adopted implicitly the frequency information, too.

Intuitively, the translation from EEG to note may be realized through a phenomenally direct mapping method, i.e., the frequency, period and amplitude (EEG) to the pitch, duration and volume (music), respectively. Some early works did follow this strategy [2]. However, as the frequency and period are reciprocally related, the generated sequence would be far from music, but an “audification” [6], in which the EEG data is adopted to control the audio signal directly.

In conclusion, we discovered a set of sonification rules for translating EEG to music based on the intrinsic nature of the both modalities. We focus in particular on scale-free phenomena, which exist widely in nature and include those of neural activity, EEG, and human behavior. Therefore, the scale-free or equivalent power-law phenomenon may be an essential mechanism of the brain. In addition, this study also addresses an old question [31]: why do people like music? A possible answer is that the brain and music both follow the same dynamic principle, the power-law, which may provide the most efficient method for humans to interact with the environment. To substantiate these speculations, much more efforts are needed for us to look into the details of the dynamics of EEG, and the neural substance of music. Furthermore, our sonification rule may provide a vivid audio window for monitoring the brain activities and may also be useful for auditory-based neurofeedback therapy in the future.

## Supporting Information

Audio S1 60s excerpt of the music from the Rapid Eyes Movement sleep.
(10.58 MB WAV)

Audio S2 60s excerpt of the music from the Slow Wave Sleep.
(10.58 MB WAV)

Audio S3 20s excerpt of the music from the White Noise.
(3.62 MB DOC)

Audio S4 20s excerpt of the music from Eyes closed.
(3.62 MB WAV)

Audio S5 20s excerpt of the music from Eyes open.
(3.53 MB WAV)

Audio S6 20s excerpt of the music from epileptic EEG.
(3.62 MB WAV)
